# An Overview of ADAM9: Structure, Activation, and Regulation in Human Diseases

**DOI:** 10.3390/ijms21207790

**Published:** 2020-10-21

**Authors:** Cheng-Wei Chou, Yu-Kai Huang, Ting-Ting Kuo, Jing-Pei Liu, Yuh-Pyng Sher

**Affiliations:** 1 Graduate Institute of Biomedical Sciences, China Medical University, Taichung 404, Taiwan; ccwei@vghtc.gov.tw (C.-W.C.); namasta.orange@gmail.com (Y.-K.H.); liualice5027@gmail.com (J.-P.L.); 2 Department of Medicine, Division of Hematology/Medical Oncology, Taichung Veterans General Hospital, Taichung 407, Taiwan; 3 Center for Molecular Medicine, China Medical University Hospital, Taichung 404, Taiwan; ttgkuo@gmail.com; 4 Chinese Medicine Research Center, China Medical University, Taichung 404, Taiwan

**Keywords:** ADAM9, biological function, inflammation, cancer

## Abstract

ADAM9 (A disintegrin and a metalloprotease 9) is a membrane-anchored protein that participates in a variety of physiological functions, primarily through the disintegrin domain for adhesion and the metalloprotease domain for ectodomain shedding of a wide variety of cell surface proteins. ADAM9 influences the developmental process, inflammation, and degenerative diseases. Recently, increasing evidence has shown that ADAM9 plays an important role in tumor biology. Overexpression of ADAM9 has been found in several cancer types and is correlated with tumor aggressiveness and poor prognosis. In addition, through either proteolytic or non-proteolytic pathways, ADAM9 promotes tumor progression, therapeutic resistance, and metastasis of cancers. Therefore, comprehensively understanding the mechanism of ADAM9 is crucial for the development of therapeutic anti-cancer strategies. In this review, we summarize the current understanding of ADAM9 in biological function, pathophysiological diseases, and various cancers. Recent advances in therapeutic strategies using ADAM9-related pathways are presented as well.

## 1. Introduction

The “a disintegrin and metalloproteases” (ADAMs) family, a subset of the zinc protease superfamily, consists of transmembrane proteins with a multidomain extracellular region, a single transmembrane sequence, and a relatively short cytoplasmic domain [1,2]. So far, around 40 family members have been identified in the mammalian genome, and among them, 22 are expressed in humans [3]. Extracellular regions of ADAMs contain several distinct domains: A prodomain followed by metalloproteinase, disintegrin, and cysteine-rich domains [4]. A majority of ADAMs—all except ADAM10 and ADAM17—also have an epidermal growth factor (EGF)-like domain, whose function is yet to be clarified [2,5,6]. ADAM proteins have been reported in numerous biological functions involving development, fertility, ectodomain shedding, cell adhesion, cell–cell interaction, vascular endothelial cell function, inflammation, immunity, signaling transduction, neurodegenerative disease, and cancer biology [1,7,8,9]. Therefore, ADAMs have important roles in diverse physiological contexts.

ADAM9 (also known as metalloprotease/disintegrin/cysteine-rich protein 9 (MDC9) or meltrin-γ), one of the ADAM proteins, was first identified in 1996 in breast carcinoma [10]. It is widely expressed in human tissues, and shows an abundant increase in pathological conditions [11]. ADAM9 expression is detected in multiple cell types, including monocytes [12], macrophages [13], neutrophils [14], keratinocytes [15], and fibroblasts [16]; and in multiple tissues, including lung [17], colon [18], kidney [19], vascular smooth muscle [20,21], nervous system [1,22], reproductive system [23], and secretary organs [24]. This indicates that ADAM9 is involved in a multitude of biological functions as well as pathophysiological conditions, such as inflammation and tumorigenesis [25,26].

In inflammation, ADAM9 contributes to monocyte fusion, mediating the conversion of monocytes-macrophages to multinucleated giant cells (MGCs) as a response to foreign bodies or bacteria; the resulting granulomatous lesions help to isolate the pathogens and also enhance phagocytotic activity [12]. ADAM9 also stimulates the inflammatory process through polymorphonuclear leukocytes, macrophages, and epithelial cells in some inflammatory diseases, such as acute lung injury or chronic obstructive pulmonary disease (COPD) [11,13,14,27]. ADAM9 is also expressed in the epidermis, where it delays wound healing by increasing collagen XVII shedding and matrix metalloproteinase-9 (MMP-9) secretion to decrease keratinocyte migration [28]. ADAM9 promotes neuropilin-1 proteolysis in the angiogenic signaling pathway of vascular endothelial cells [29]. ADAM9 has been demonstrated to be involved in neurodegenerative disease by regulating the cleavage of amyloid precursor protein (APP), which might be relevant in Alzheimer’s disease [4,30,31]. ADAM9 was also shown to be involved in myogenesis and formation of myotubes, which might contribute to the endocardial cushion during embryonic development [32]. Recently, ADAM9 was reported to shed the interleukin-11 receptor, which is involved in inflammatory conditions, bone homeostasis, hematopoiesis, and fertility [33]. Altogether, ADAM9 is expressed broadly in human tissues, and participates in the development, inflammatory processes, and degenerative diseases.

Despite its wide distribution in mammalian tissue and its regulatory function in development, the *Adam9*^−^/^−^ mouse model showed no apparent morphological changes or histopathological defects during development and adult life [34]. Therefore, the effects of ADAM9 deficiency might be compensated by other ADAM family members, but no further investigation to address the potential ADAM family members [21]. However, subsequent animal studies demonstrated that the deletion in the ADAM9 induced photoreceptor degeneration of both retinal rods and cones, resulting in visual acuity impairment in young canine models. Malformation of retinal pigment epithelium, disrupted contact with photoreceptor outer segments, and abnormal gaps between retinal layers were demonstrated, leading to the degeneration of the retina [35,36]. These mice showed less ocular neovascularization than wild-type mice during the pathological neovascularization, which was not associated with developmental retinal angiogenesis. Furthermore, ADAM9 null mutations were shown to cause retinal degeneration in human patients, leading to cone–rod dystrophy (CRD) [36]. In the opposite direction, overexpression of ADAM9 enhances angiogenesis by increasing the shedding of several angiogenesis-related endothelial membrane proteins including Tie-2, vascular endothelial growth factor receptor-2, VE-cadherin, ephrin type B receptor 4 (EphB4), CD40, and vascular cell adhesion molecule 1 (VCAM-1) [37]. Collectively, during development, lack of ADAM9 seems to affect mainly the visual system, whereas other biological functions might be compensated by other ADAMs.

## 2. Structure and Molecular Function of ADAM9

ADAM9 is composed of several domains that have their specific functions, including protease activation and substrate recognition.

### 2.1. ADAM9 Structure

**Figure 1:**
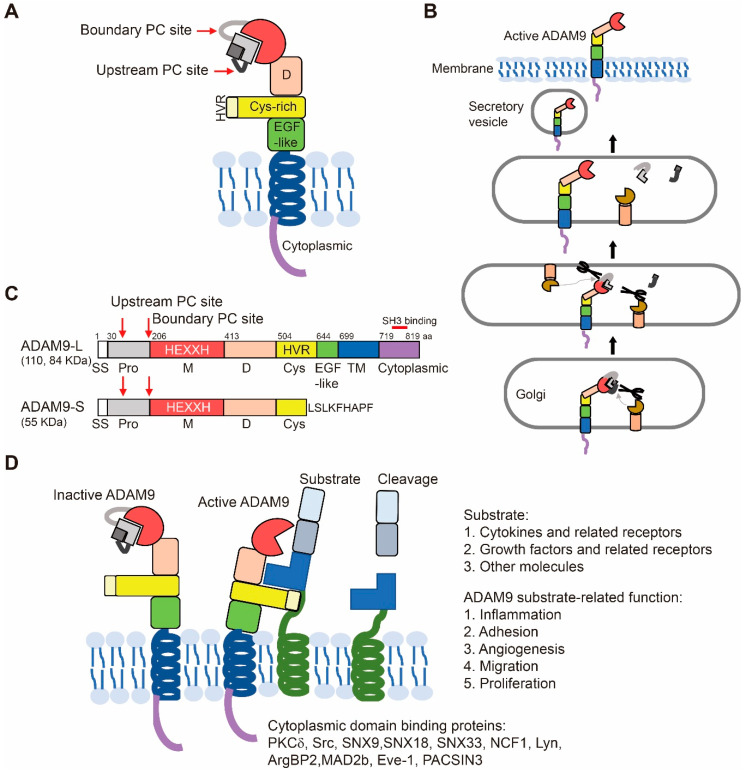
Characterization of a disintegrin and metalloprotease 9 (ADAM 9). (**A**) The organization of the C-shape structure when anchored to the membrane is shown. (**B**) ADAM9 is activated during transport via Golgi bodies to the cell membrane by removal of the inhibitory pro-domain. The pro-domain cleavage is performed at the two PC sites by furin or proprotein convertase. (**C**) Schematic diagrams of the domain structure of the long (ADAM9-L, 110 and 84 KDa) and short (ADAM9-S, 55 KDa) forms of ADAM9. ADAM9-S lacks exon 12 from the ADAM9-L genetic sequence. aa, amino acid. SS, signal sequence. Pro, pro-domain. M, metalloprotease domain. D, disintegrin domain. Cys, cysteine-rich. TM, transmembrane. PC site, proprotein convertase (PC) consensus cleavage site. HVR, hyper-variable region. (**D**) Active ADAM9 recognizes its substrates via the HVR of the cysteine-rich domain and releases the extracellular fragments of membrane-bound cytokines and growth factors. It also cleaves receptors and other molecules for signal transduction.

The domain structure of ADAM9, the process of ADAM9 activation, and the two isoforms of ADAM9 are shown (Figure 1A–C).

#### 2.1.1. Pro-Domain and Its Cleavage for Protease Activation

ADAM9 contains a signal sequence at its N-terminus (from aa 1 to aa 29) that guides the secretory pathway of ADAM9 to traffic to the cell surface, and the transmembrane domain of ADAM9 anchors it on the cell membrane (Figure 1B). ADAMs are biosynthesized as zymogens with the pro-domains preserving their inactive state. The pro-domain of ADAM9 is located from aa 30 to aa 205 and is cleaved by the furin-like proprotein convertase (PC) (Figure 1A,B).

Pro-domains of ADAMs can serve as inhibitors of ADAM catalytic activity. Recombinant pro-domains of ADAM9 [30], ADAM10 [38], and ADAM17 [39], purified from *Escherichia coli*, have been demonstrated as competitive inhibitors against their proteolytic activities in vitro. ADAMs (ADAM9, ADAM10, and ADAM17) contain two distinct PC cleavage sites within the pro-domain, including the upstream site and the boundary site between the pro- and the catalytic domain. The upstream site plays an important role in the activation of the ADAM proenzyme. Without cleavage at the upstream PC site, the ADAM protein might exist in the proenzyme-like form on the cell surface [40]. Thus, the processing of the pro-domains is responsible for the activation of ADAM9 to regulate cell signaling [2]. Meanwhile, meprin β, cleaving at the pro-domain of ADAM9, 10, and 17, could further increase ADAM protease activities [41].

#### 2.1.2. Metalloproteases, Disintegrin, Cysteine-Rich, EGF-Like, and Transmembrane Domains

ADAM9 contains the consensus sequence for the catalytic Zinc-binding motif of metalloproteases (HExGHxxGxxHD) within its metalloprotease domain (aa 206–412), which can be catalytically active. The name of the disintegrin domain comes from its presence in the snake venom metalloproteases, which are involved in the binding of platelet integrin receptors. The disintegrin domain of ADAM9 is ~90 aa long, located on aa 413–503. The domain serves as a ligand to interact with multiple β1 renal integrins and therefore could regulate attachment to the critical components of the extracellular matrix (ECM) [2,19]. The cysteine-rich domain of ADAMs is capable of binding ECM independent of the disintegrin-integrin interaction. It has been reported to be involved in membrane fusion. In ADAM9, the function of the cysteine-rich domain is not yet demonstrated. In ADAM13, the cysteine-rich domain cooperates intramolecularly with its metalloprotease domain to regulate its function in vivo [42]. The tandem disintegrin and cysteine-rich domains of ADAM13 are able to bind to both ECM proteins like fibronectin and laminin and to activated β-1-containing integrins on the cell surface. The cell–cell adhesion of fibroblasts and melanoma cells is significantly reduced when incubated with the recombinant tandem disintegrin and cysteine-rich domains of ADAM9 [42]. The recognition of other proteins by these extracellular domains of ADAM9 may represent a mechanism how this protease can select and hold on to its substrates [43].

The function of the EGF-like domain has yet to be fully understood. The transmembrane domain anchors the protein to the cell membrane. ADAM9 was found to co-localize with VE-cadherin at cell–cell junctions in confluent endothelial monolayers. In addition, ADAM9 must be expressed on both adjacent cells to localize to the cell–cell junctions, indicating the ability to self-associate through ectodomain interactions [9].

#### 2.1.3. Cytoplasmic Domain

The cytoplasmic domain of ADAM9 plays a role in regulating its catalytic activity. The catalytic activity of ADAMs was reported to have a strong correlation with the Src-homology 3 (SH3) domain binding to the cytoplasmic domain. The majority of ADAM-interacting SH3 proteins are sorting- and endocytosis-associated adapter proteins or kinases. The cytoplasmic domain of ADAM9 contains 4 potential SH3 binding sites (aa 746–759, aa 754–767, aa 785–798, and aa 802–816) and interacts with SH3 domain-containing proteins, such as SNX33 (Sorting nexin-33), SNX9 (Sorting nexin-9), tyrosine protein kinase Tec, SNX18 (Sorting nexin-18), neutrophil cytosol factor 1, tyrosine protein kinase Lyn, and ArgBP2 (Sorbin and SH3 domain-containing protein 2) [44].

SNX9 is involved in endocytosis and intracellular vesicle trafficking [45]. In the *SNX9* knockdown breast cancer cells, total ADAM9 protein expression and cell surface ADAM9 proteins are elevated, and the protease function of ADAM9 is active in shedding of the ADAM9 substrate EphB4. This indicates that SNX9 limits ADAM9’s proprotein processing during the secretory process to control the cell-surface levels of ADAM9. Therefore, SNX9 serves as an important intracellular trafficking regulator of ADAM9 [46]. In addition, the binding of protein kinase C δ (PKC δ) to the cytoplasmic domain of ADAM9 increases the ectodomain shedding of the proHB-EGF (heparin-binding EGF-like growth factor) [47]. Taken together, the cytoplasmic domain of ADAM9 has a role in regulating the function of ADAM9, depending on the interacting proteins.

#### 2.1.4. Two Isoforms of ADAM9

Two alternatively spliced ADAM9 transcripts are expressed in the form of a transmembrane protein (ADAM9-L) and a secreted isoform (ADAM9-S) (Figure 1C). Fry et al. demonstrated that ADAM9-L suppresses cell migration in BT-549 breast cancer cells independent of its metalloproteinase activity, whereas ADAM9-S promotes cell migration in a manner requiring its metalloproteinase activity [48]. ADAM9-S is a secreted protein, lacking the transmembrane and cytoplasmic domains via excising exon 12 from the ADAM9 mRNA, and contains 8 unique amino acids (LSLKFHAPF) not present in ADAM9-L. ADAM9-S promotes cancer cell migration and invasion via its metalloprotease activity and is also able to bind directly to α6β4 and α2β1 integrins on the cell surface through the disintegrin domain [49].

### 2.2. Substrate Recognition and Cleavage

Crystal structures of ADAMs (ADAM10 and ADAM17) have provided exciting details about the mechanisms of substrate recognition and cleavage by ADAMs [50,51]. These structures have demonstrated the C-shaped structure of ADAMs, which facilitates their function in substrate recognition and cleavage. The C-shaped arm includes the metalloproteinase, disintegrin, and cysteine-rich domains and the hypervariable region (HVR) (Figure 1A). This structure mediates the functions of both the proteolytic and the adhesion domain, which regulates target recognition. Moreover, the HVR also regulates cell–cell and cell–matrix interactions in proteolytically inactive ADAMs [4,52,53].

Reported substrates for ADAM9 shedding are briefly summarized in three groups (Figure 1D): (I) Cytokines and related receptors: CD40 [54], interleukin-11 receptor [33], and tumor necrosis factor-α (TNF-α) [55]. (II) Growth factors and related receptors: pro-HB-EGF [47], insulin-like growth factor binding protein-5 [56], p75 neurotrophin receptor, insulin B chain, pro-EGF, FGF receptor 2 iiib, EphB4 [7], and neuregulin 1β [54]. (III) Other molecules: ADAM10 [30], Delta-like 1 [57], angiotensin-1 converting enzyme [58], APP, Kit-ligand, collagen XVII [7], laminin [49], fibronectin, gelatin [54], Tie-2 [37], VE-cadherin, vascular endothelial growth factor receptor-2, and vascular cell adhesion molecule 1 [54]. Despite ADAM9 shares a number of the mentioned substrates with ADAM10 and ADAM17, they still have some different functions in biology, which might be due to the unique substrates by each protease. A study demonstrated that ADAM10 and ADAM17 have different amino acid favors at multiple positions surrounding the substrate cleavage site by peptide library screening and analysis of individual consensus substrates [59]. Furthermore, there have been no reported studies of the cleavage site specificity of ADAM9. It is still unclear whether ADAM9 has its unique amino acid sequence near the substrate cleavage sites, distinct from that of ADAM10 or ADAM17. No relevant research shows which is the main sheddase under specific conditions. Based on this list, ADAM9-mediated substrate shedding is capable of stimulating a variety of functions in inflammation, adhesion, angiogenesis, migration, and proliferation, and we would expect that the dysregulation of ADAM9 promotes inflammatory-related diseases and malignancy. Although most substrates were identified in vitro, it is still unclear for many substrates whether they are in fact cleaved in vivo. Interestingly, in vivo cleavage-independent pathway of ADAM9 plays a crucial role in tumorigenesis. We will discuss the related function in Section 6.

## 3. Pathological Roles of ADAM9 in Neurodegenerative and Retinal Diseases

Since ADAM9 is widely expressed in the human body and regulates a variety of biological functions, ADAM9 also plays an important role in pathological diseases including degenerative, retinal, inflammatory and tumor biology. In the following sections, we will briefly summarize the role of ADAM9 in different disease entities.

### 3.1. ADAM9-Mediated Neurodegenerative Diseases

Although ADAM9 is not crucial for development and survival, ADAM9 is still involved in various human pathologic states, including neurodegenerative disorders, vascular diseases, and inflammatory diseases [21]. Alzheimer’s disease, a common neurodegenerative disorder, presents with progressive dementia and cognitive disorders. It is characterized by amyloid senile plaques in the vasculature of the brain and is due to the accumulation of amyloid beta-peptides (Aβ peptides) by β-secretase 1 (BACE1) and γ-secretase cleavage of APP [60]. Normally, α-secretase would cleave the majority of APP and prevent β-secretase 1 from cleaving APP to form amyloidogenic peptides. Among Alzheimer’s disease patients, decreased levels of APP processed by α-secretase have been reported [61]. ADAM9, ADAM10, and ADAM17 belong to the family of α-secretases that cleaving APP to reduce Aβ peptide production. Among these ADAMs, ADAM10 is probably the most relevant sheddase for APP. However, promoter polymorphisms of ADAM9 have been reported to have a significant association with Alzheimer’s disease [62]. Therefore, the increase of α-secretase activity seems to be a therapeutic strategy against Alzheimer’s disease [22,60].

On the contrary, in SY5Y neuroblastoma cells overexpressing APP, mimicking Alzheimer’s disease, treatment with ADAM9 pro-domains inhibited the activity of ADAM9 but reduced the production of Aβ peptides. Despite the fact that ADAM9 is an α-secretase and expected to reduce the Aβ peptides, however, ADAM9 can cleave another α-secretase, ADAM10. Inhibiting ADAM9 protease function reduced the shedding of ADAM10 and subsequently increased membrane-bound ADAM10 to generate higher levels of soluble APP through α-secretase cleavage and decreased Aβ levels [30]. Therefore, increased α-secretase activity at the cell membrane through different molecular mechanisms may have a beneficial effect in Alzheimer’s disease.

ADAM9 also mediates the shedding of the cellular prion protein (PrPc) involved in neurodegeneration. ADAM9, found in different cell types, enhances PrPc shedding in an ADAM10-dependent manner, but not through direct cleavage of the PrPc. Although ADAM9 might mediate ADAM10 shedding, under different biological conditions, the proportion of soluble ADAM10 versus membrane-bound ADAM10 and the impacts on substrate shedding is yet to be determined [1].

### 3.2. ADAM9-Mediated Retinal Diseases

CRD, which is a hereditary retinal disease affecting photoreceptor function, reduces visual acuity and results in color-vision abnormalities [36]. Several genes have been shown to be involved in CRD. The loss of the ADAM9 was also related to CRD in humans [35]. Meanwhile, retinopathy of prematurity in infants, diabetic retinopathy, and age-related macular degeneration are all characterized by ocular neovascularization, one common cause of blindness. A murine model of retinopathy of prematurity showed that ADAM8, 9, and 10 were related to oxygen-induced retinopathy, which is a kind of pathological neovascularization as well [63].

### 3.3. ADAM9-Mediated Vascular Disease

The association between pathological neovascularization and ADAM9 had been demonstrated. For example, ADAM9 knockout mice have reduced pathological neovascularization compared to control mice [37]. In addition, ADAM9 overexpression increased the ectodomain shedding of adhesion molecules and membranous proteins from endothelial cells and aggravated the pathological neovascularization [37,64]. With evidence showing that other ADAMs (ADAM10 and ADAM17) play roles in the development of abdominal aortic aneurysm [65,66], a recent study demonstrated that ADAM9 expression was up-regulated in a murine abdominal aortic aneurysm model. ADAM9, modulated by miR-126, could induce inflammatory cascades and the infiltration of macrophages into the aortic wall [20]. Additionally, knockdown of ADAM9 suppressed aneurysm formation through PI3K/AKT/NF-κB pathway inhibition, an activated pathway in the murine aneurysm model [67]. Therefore, ADAM9 can induce retinal disorders as well as pathological vascular diseases.

## 4. ADAM9 in Infectious and Inflammatory Diseases

### 4.1. Infection and Tissue Damage

Encephalomyocarditis virus (EMCV) causes myocarditis or encephalitis in animals. Recent studies demonstrate that ADAM9 is required for the entry phase of EMCV infection, independent of its metalloprotease activity [68]. Using a CRISPR-Cas9 screen, another group also identified that ADAM9 is an essential factor for the early stages of both human and murine EMCV infection [69]. Moreover, other studies showed that ADAM9 transcript in the blood cells was up-regulated and might serve as a biomarker representing tissue damage by infection, inflammation, and tissue injury [11].

### 4.2. Lung Injury and COPD

In neutrophils, the ADAM9 proteins are mainly located in the tertiary, gelatinase granules and the secretory vesicles. Several proinflammatory agonists (fMLP, IL-8, and TNF-α), which induce degranulation of PMNs, also increase surface ADAM9 levels on PMNs in a concentration-dependent manner. Up-regulation of ADAM9 was found in acute lung injury in mice, in addition to more proteolysis of ECM proteins. Therefore, ADAM9 regulates this process and promotes the acute lung injury [14]. Increased ADAM9 levels were also found in COPD patients. The role of ADAM9 in COPD has been shown to involve emphysema development and airway disease in mice. Therefore, targeting ADAM9 might be a therapeutic choice for COPD patients [13].

### 4.3. Chronic Wounds

Since ADAM9 regulates cell–cell and cell–matrix interactions, it is presumably also involved in the migration and proliferation of keratinocytes during the wound healing process. It seems that ADAM9 mediates collagen XVII shedding to influence keratinocyte migration in wound repair. In an ADAM9 knockout mouse model, researchers saw the increased distribution of collagen XVII on the basolateral keratinocytes for promoting keratinocyte migration in wound healing, and the effect was reversed by adding recombinant ADAM9 [28]. This might be due to the recombinant soluble ADAM9 that contained the catalytic activity. Therefore, the increased expression of ADAM9 in wound repair has a negative impact on re-epithelialization.

## 5. Role of ADAM9 in Cancers

ADAM9 participates in the regulation of various tumor processes. In addition to metastasis, ADAM9 also plays an important role in tumor proliferation, angiogenesis, and even immune evasion (Table 1).

### 5.1. Lung Cancer

In lung cancer, the dysregulation of ADAM9 was documented long ago. Various studies demonstrate that ADAM9 is a major player in lung cancer progression and metastasis [25,26]. Studies show the overexpression of ADAM9 shortens overall survival, and the same pattern was found in various cohorts by different groups [70,71,72]. By immunohistochemical staining, high protein expression of ADAM9 was found to be correlated with a poor 5-year survival rate in an Asian cohort (10/17, 59%) [25], and in resected stage I lung cancer (29/63, 46%) [73,74].

Metastasis is the leading cause of lung cancer-related death, and nearly 50% of late-stage patients with lung cancer exhibit brain metastasis. ADAM9 has been reported as a major player in several steps of this process [91]. Shintani et al. first described that overexpression of ADAM9 promotes the adhesion of tumor cells to vascular endothelial cells, which suggests the importance of ADAM9 during metastasis. In addition, ADAM9 enhances cell migration and anoikis resistance to promote metastasis by a novel mechanism. Silencing ADAM9 down-regulates the RNA expression of CUB domain-containing protein 1 (CDCP1) and tissue-type plasminogen activator (tPA) but up-regulates the expression of plasminogen activator inhibitor-1 (PAI-1) [25]. Moreover, ADAM9 enhances the activity of tPA to cleave CDCP1, resulting in CDCP1 activation that promotes metastatic processes of cell migration and anoikis resistance. Thus, ADAM9 promotes the CDCP1 activation for lung cancer metastases to the brain through a tPA-based pathway.

This ADAM9-CDCP1 axis to lung cancer metastasis is also validated in several other reports as well [92,93]. The ADAM9-CDCP1 axis was confirmed to be necessary for lung cancer cell migration and survival in vitro and in vivo; and moreover, the authors demonstrated that ADAM9 decreases the expression of miR-1 and miR-218, which target the 3′-UTR of CDCP1 to suppress its expression. Thus, ADAM9 promotes the elevated protein levels of CDCP1. To achieve brain metastasis, the disruption of the blood–brain barrier is necessary for tumor cell entry. ADAM9 also participates in this process by up-regulating angiopoietins 2 and tPA. Silencing ADAM9 enhances the membrane expression of VE-cadherin, which is responsible for maintaining the restrictive barrier between endothelial cells and reduces the cell permeability of endothelial cells in vitro [26]. These findings illustrate the multiple roles of ADAM9 in lung cancer metastasis.

The essential role of angiogenesis in tumor progression is well-defined in numerous cancer types, including lung cancer, and chemical stimulation performed by various angiogenic proteins is crucial and necessary. Meanwhile, the conditioned medium from ADAM9-silenced cells suppresses tube formation of human umbilical vein endothelial cells; moreover, silencing ADAM9 inhibits angiogenesis in vivo. By angiogenesis antibody array and further ELISA, a previous study identified that ADAM9 mediates the expression of angiogenesis factor, interleukin 8 (IL-8). And IL-8 is known to bind and then activate its high-affinity receptor, C-X-C Motif Chemokine Receptor 2 (CXCR-2). Moreover, the neutralizing antibody of CXCR-2 reverses the ADAM9-mediated HUVAC tube formation. These evidences suggest a possible mechanism of ADAM9-mediated angiogenesis through the IL-8-CXCR2 axis [72]. In another study, vascular endothelial growth factor, a well-known angiogenic protein, was down-regulated in the ADAM9-silenced cell-conditioned medium [26]. Taken together, these results suggest that ADAM9 participates in tumor angiogenesis by increasing the activity of various angiogenic proteins in lung cancer.

Micro-RNAs (miRNAs), such as miR-425, miR-488 and miR-590, have been reported as regulators of ADAM9 in lung cancer. Via prediction tools and luciferase reporter assay, the 3′-UTR of ADAM9 is identified as the target sites of 3 micro-RNAs to down-regulate ADAM9 mRNA expression [70,94,95].

### 5.2. Prostate Cancer

Fritzsche et al., demonstrated that both mRNA and protein overexpression of ADAM9 is correlated with poor relapse-free survival of prostate cancer [75]. By immunohistochemistry, more than 60% of recurrent prostate tumors have elevated protein expression of ADAM9 [76].

The progression and growth of prostate cancer is dependent on androgens; thus, androgen deprivation by castration or target therapy has become the predominant therapies for advanced prostate cancer. However, tumors treated in this way develop into a more aggressive and castration-resistant type, called androgen-independent prostate cancer (AIPC). Lin et al., uncovered the mechanism of maintaining ADAM9 protein stability in AIPC. N-α-Acetyltransferase 10 protein (Naa10p) has been identified as an oncoprotein in prostate cancer [77]. Silencing Naa10p down-regulates the protein expression of ADAM9 to suppress tumor growth and metastasis in vitro and in vivo. Silencing Naa10p also accelerates ADAM9 protein degradation, and the direct interaction between Naa10p and ADAM9 has been confirmed by co-immunoprecipitation. Taken together, the protein stability of ADAM9 is highly maintained by Naa10p to drive AIPC tumor outgrowth and metastasis. And this Naa10p-mediated stabilization of ADAM9 maybe exist in other cancer types to drive tumorigenesis as well.

ADAM9 regulates prostate cancer progression and outcome in other ways as well. Silencing ADAM9 impairs the endocytosis of integrin β1 to increases its expression on cell membrane to enhance the integrin-mediated cell adhesion and suppress cell migration in vitro. Notably, metalloproteinase inhibitors (Batimastat or GM6001) cannot reverse the integrin β1 expression or integrin-mediated migration effects, which suggests the proteolytic activity of ADAM9 is dispensable for this mechanism. In contrast, the direct interaction between ADAM9 and integrin β1 has been confirmed by co-immunoprecipitation, and both proteins co-localize on early endosomes in vitro. These findings suggest ADAM9 mediates integrin β1 stability in a non-catalytic manner [78].

MiRNAs also play a role in regulating ADAM9 in prostate cancer, especially miR-126. Hua et al. identified the binding site and the effect of miR-126 on reducing ADAM9 expression via luciferase reporter assay. And silencing ADAM9 by miRNA has similar inhibitory effects on cell proliferation, migration, and invasion in vitro as did miR-126 overexpression [76].

### 5.3. Liver Cancer

Several pieces of evidence suggest the overexpression of ADAM9 contributes to poor patient outcomes and lower response to immune checkpoint blockade therapy [79]. Kohga et al. identified MHC Class I polypeptide-related sequence A (MICA), which is a ligand on cancer cells to elicit attack by natural killer cells, as a novel target of ADAM9. Membrane-bound MICA (mMICA) can be cleaved by ADAM9 to release soluble MICA (sMICA) by ADAM9 as an immunological decoy to suppress immune surveillance. The knockdown of ADAM9 up-regulates the expression of mMICA on the cell membrane and down-regulates sMICA in culture supernatant in vitro [80].

Metastasis is also an urgent issue in liver cancer therapy. IL-6 is a major mediator of invasion and metastasis in liver cancer [81,96]. Dong et al. have demonstrated that IL-6 enhances ADAM9 expression through activating the JNK pathway in vitro. Silencing ADAM9 not only reduces the primary tumor size but also suppresses the metastasis rate to lung; in contrast, overexpressing ADAM9 accelerates primary tumor growth and promotes the metastasis to the lung [81].

Numerous studies identify the negative regulation of ADAM9 by miRNAs in liver cancer. MiR-126 [97]. miR-203 [98], and miR-488 [99], target the 3’-UTR of ADAM9 and down-regulate ADAM9 to suppress cell migration and invasion in vitro.

### 5.4. Breast Cancer

In breast cancer, the expression of ADAM9 is up-regulated compared to normal tissue [82]. ADAM9 also contributes to disease progression by promoting tumor extravasation and migration ability [83]. An upstream role of ADAM9 in the trans-endothelial migration pathway was suggested by Micocci et al. Knockdown of ADAM9 down-regulates the mRNA expression of ADAM15 and MMP2 but not ADAM10, ADAM17, or MMP9 in vitro [100].

Triple-negative breast cancer (TNBC) is the most aggressive type of breast cancer. In some subtypes of TNBC, methylation deregulation causes the overexpression of EGFR to enhance cell proliferation and survival. ADAM9 shares EGFR’s methyltransferase, according to chromatin precipitation assays, and nuclear receptor-binding SET domain protein 2, a member of the histone methyltransferase family, up-regulates the expression of both ADAM9 and EGFR and promotes TNBC cell resistance to EGFR inhibitors [84].

Several miRNA expression profiles and target sites on ADAM9 have been studied in breast cancer, including miR-126 [101,102], miR-154 [103], and miR-33a [104]. The binding sites of these miRNAs are located on 3′-UTR of ADAM9, and lower expression of these miRNAs results in the overexpression of ADAM9 and promotes cancer cell migration and invasion in vitro.

### 5.5. Pancreatic Cancer

In recent years, several studies have suggested that elevated mRNA expression of ADAM9 shortens the overall survival of pancreatic cancer patients [85], a finding that was confirmed by immunohistochemistry [24]. Increased ADAM9 expression also correlates with higher tumor grade and progression [86].

KRAS signaling is necessary to maintain tumorigenesis in pancreatic cancer. Yuan et al. discovered that dysregulated KRAS signaling enhanced the expression of ADAM9 via NF-kB cascade [87]. Notably, the knockdown of ADAM9 suppresses the downstream pathway of KRAS and MEK-ERK signaling as well [24]. Taken together, a feedback loop between ADAM9 and KRAS is implied.

Circular RNA, a type of single-stranded RNA produced by non-canonical linear splicing, called back-splicing, takes on a covalently closed-form in the cytoplasm and regulates biological function by acting as a micro-RNA or protein inhibitor [105]. Studies have demonstrated that circular ADAM9 (circ-ADAM9) is up-regulated in pancreatic cancer cells and is correlated with poor prognosis. Circ-ADAM9 absorbs and inhibits miR-127, which is a tumor suppressor. Overexpressing circ-ADAM9 increases ERK signaling to promote cell proliferation and migration in vitro, and silencing circ-ADAM9 delays pancreatic tumor growth in vivo [88].

ADAM9 is also regulated by various miRNAs in pancreatic cancer. MiR-489 [87], miR-126 [106,107], and miR-502f [85] target the 3′-UTR of ADAM9 and down-regulate ADAM9 directly to suppress cell migration and invasion in vitro.

### 5.6. Glioma

Glioma causes nearly 8% of all cancer-related deaths every year, and glioblastoma (GBM) is an advanced and aggressive glioma that has only a 10% 5-year survival rate. Based on the RNA-seq data from 303 glioma patients, the elevated mRNA expression of ADAM9 is correlated with poor progression-free survival and overall survival [89]. In addition, both mRNA and protein expression of ADAM9 are up-regulated in GBM patients and correlated with short overall survival in different cohorts as well [90].

Tumor invasion highly depends on the interaction between the ECM and tumor cells for most cancer types. Tenascin-C (TNC), a major component of the ECM, activates the JNK pathway to promote tumor invasion in GBM. One study demonstrated that both mRNA and protein expression of ADAM9 are up-regulated in TNC-treated GBM cells [90]. Moreover, treatment with JNK inhibitor (SP600125) inhibited TNC-induced ADAM9 expression, and silencing ADAM9 in TNC-treated GBM cells suppressed cell migration and invasion.

ADAM9 regulation by miRNAs is also well-studied in glioma. MiR-543 [108] and miR-140 [109] are two down-regulated miRNAs in GBM tumor tissue. Similar to other miRNAs in various tumor types, the binding sites are located in the 3′-UTR of ADAM9. Overexpression of ADAM9 reverses the inhibitory effects of miR-543 and miR-140 on cell proliferation, migration, and invasion of GBM cells in vitro.

### 5.7. Other Cancers

For patients with advanced tumors, chemotherapy is the most commonly and last used treatment, even those with cancer types known to be non-response to chemotherapy. Recently, several studies demonstrate the necessity of ADAM9 in chemo-resistance in distinct cancer types. Josson et al. first demonstrates the inhibition of ADAM9 enhances the sensitivity to numerous common chemotherapy drugs (including doxorubicin, cisplatin, and gemcitabine) in prostate cancer cells [110]. This suggests that ADAM9 participates in the chemo-resistance in tumor progression. Ueno et al., found that silencing ADAM9 induces apoptosis in cisplatin-resistance ovarian cancer cells through impaired EGFR signaling; moreover, the treatment of neutralizing antibody of ADAM9 has the same inhibitory effect to cisplatin-resistance ovarian cancer cell as well [111]. Fu et al. found the up-regulated protein expression of ADAM9 and EGFR in 5-FU-resistance colon cancer cell lines, and ADAM9 silencing by micro-RNA re-sensitized the colon cancer cells to 5-FU [18]. These evidences conclude the possibility of ADAM9 mediating chemoresistance in the advanced tumor stage. Therefore, the combined therapies with the inhibition of ADAM9 and chemotherapy may be feasible in clinical treatment.

## 6. ADAM9 in the Tumor Microenvironment

ADAM9 is not only reported to be up-regulated in tumor cells but also in the tumor microenvironment, which contains blood vessels, immune cells, and ECM. Interactions between these components of the microenvironment enhance tumor metastasis and epithelial–mesenchymal transition (EMT).

### 6.1. Neutrophil

ADAM9 proteins are stored in granules and secretory vehicles of human and murine polymorphonuclear neutrophils (PMNs) [14]. Minimal quantities of surface ADAM9 proteins are detected in unstimulated PMN, but surface ADAM9 levels are rapidly increased in activation of PMN with degranulating agonists. Activated neutrophils can express both membrane-bound and soluble ADAM9 to promote extracellular matrix protein degradation during acute lung injury. In addition, the recombinant disintegrin domain of ADAM9 can interact with integrins to activate neutrophils, which further induces chemotaxis, production of reactive oxygen species, neutrophil extracellular trap formation, and delays the spontaneous apoptosis of neutrophils [112]. That implies the interaction between ADAM9 and integrins is important to activate neutrophils. Given that tumor cells express both membrane-bound and soluble ADAM9, it is expected that could promote neutrophil activation in the tumor microenvironment. Moreover, several studies have shown that neutrophil extracellular traps attract cancer cells and form distant metastases in the lung or liver in several mouse tumor models [113]. These studies suggest ADAM9-mediated neutrophil activation, either ADAM9 from cancer cells or neutrophils, contributes to tumor progression.

### 6.2. Monocytes/Macrophages

In chronic inflammation, macrophages fuse with other macrophages to form MGCs, which are stimulated by CD98, RANKL, and MCSF. ADAM9 is reported to be involved in CD98-mediated and RANKL-mediated monocytes fusion [12]. MGCs are the main components of granulomas, which are known to limit tuberculosis infection. Currently, there is no reported study showing the role of ADAM9 in osteoclast formation. In tuberculosis patients, lipomannans is one of the major lipoglycans embedded within the mycobacterial envelopes, and it can induce large MGC assembly, which is mediated by TLR2 and the β1 integrin/ADAM9 cell fusion machinery [114]. Moreover, in giant cell tumors of bone and aneurysmal bone cysts, studies have demonstrated the presence of MGC-rich lesions of neoplastic origin. Giant cell tumors of bone are osteoclast-like giant cells and display locally aggressive behavior. Therefore, ADAM9 might have roles in regulating giant cell tumors [115].

### 6.3. Platelet

**Figure 2:**
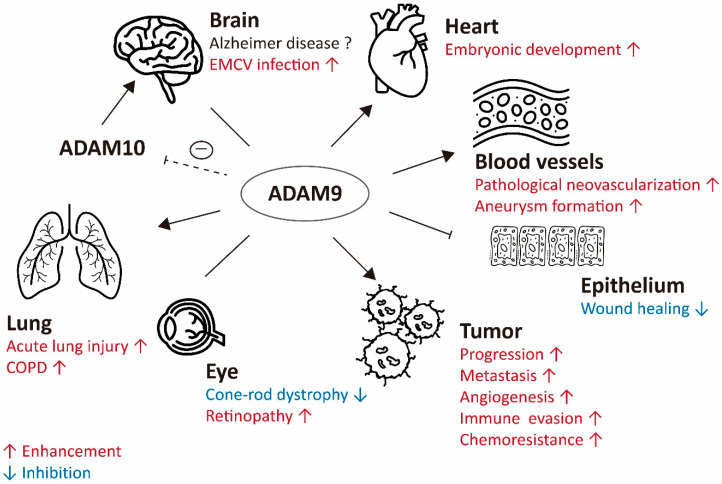
The role of ADAM9 in various pathophysiological conditions. Brain: ADAM9 acts as an α-secretase to reduce the risk of Alzheimer’s disease, but it can reduce the proportion of membrane-anchored ADAM10 and results in reduced α-secretase activity of ADAM10. Therefore, ADAM9 has inconclusive effects on brain. ADAM9 enhances the EMCV entry. Heart: ADAM9 is active in embryonic heart development. Blood vessels: ADAM9 enhances the pathological neovascularization and aneurysm formation. Epithelium: ADAM9 delays chronic wound healing. Tumor: ADAM9 promotes tumor progression, metastasis, angiogenesis, and immune evasion. Eye: ADAM9 increases retinopathy and loss of ADAM9 relates to CRD. Lung: ADAM9 is up-regulated in acute lung injury and promotes the development of COPD. Dashed T-bar arrow, inhibiting ADAM9 protease function reduced the shedding of ADAM10; T-bar arrow, inhibition of wound healing; Enhancement in red; Inhibition in blue.

Platelets can bind to tumor cells to form a physical shield to prevent immune cell attack and promote EMT/metastasis through TGF-β and NF-κB signaling [116,117,118]. Various types of integrins are found on the surface of platelets, including integrin α6β1. Lack of integrin α6β1 on platelets suppresses tumor metastasis to lungs in breast cancer and melanoma, and integrin α6β1 deficiency or treatment with integrin α6β1 neutralizing antibody impairs the interaction between platelets and tumor cells in breast cancer and colon cancer. On the other hand, the low expression of ADAM9 on tumor cells also impedes the interaction between platelets and tumor cells in breast cancer and melanoma. The interaction between ADAM9 and integrin β1 has been proven in several studies, which suggests that ADAM9 may have a role in platelet-mediated metastasis [119] (Figure 2).

## 7. Therapeutic Potential of Manipulating ADAM9 Expression in Cancer Treatment

ADAM9 is strongly implicated in several pathological processes including inflammation and tumor progression. Certain types of environmental stress can increase ADAM9 expression, which might stimulate the mechanistic adaptation of tumor cells to adverse conditions and help cancer cells to survive. Moreover, ADAM9 overexpression is associated with poor cancer patient outcomes. Therefore, ADAM9 can be considered a potential therapeutic target for dealing with ADAM9-mediated cancers.

### 7.1. MicroRNA and Proteins Targeting ADAM9

Several miRNAs have been reported to directly target ADAM9 mRNA and further reduce ADAM9 expression in cancer cells as previously mentioned. Protein shedding is the major function of ADAM9 and requires other domains to recognize the substrates. Moreover, ADAM9 has the protease-independent function to mediate cell adhesion and contribute in promoting cancer metastasis. Theoretically, completely reducing ADAM9 protein levels is considered to effectively interrupt its function rather than inhibiting its catalytic activity. ADAM9 silencing, either by siRNA or shRNA, has demonstrated to significantly reduce cancer progression in many cancer types. In addition, *ADAM9*-targeting miRNA, although targeting other genes, would have potential therapeutic effects in cancer treatment. Several miRNA-based therapies among different cancers have been conducted and this strategy would have potential clinical implications in cancer treatment [120]. The typical one is miR-126, which is down-regulated in multiple cancer types, including pancreatic cancer [107], breast cancer [101,102], and hepatocellular carcinoma (HCC) [97]. Other miRNAs, targeting ADAM9, such as miR-203 [98], miR-488 [99], miR-154 [103], miR-33a [104], miR-489 [87], and miR-502f [85] were reported in reduced levels in cancer cells. Overexpression of these miRs suppressed ADAM9 expression and reduced cancer cell invasion/metastasis.

Two proteins were designed to influence ADAM9’s protease activity. One is the mouse prodomain of ADAM9 (proA9; amino acids 24–204), which can block the protease region as a competitive inhibitor of human ADAM9 [30]. The other is an ADAM9 antibody, which can recognize the protease domain, and has been reported to inhibit the gastric cancer progression [121].

### 7.2. MMP Inhibitors

Several reagents have been reported to influence ADAM9 expression or function by direct or indirect effects. CGS27023 (small molecule, Novartis) and batimastat (BB-94, hydroxamate derivative, Sigma-Aldrich, St. Louis, MO, USA) are broad-spectrum MMP inhibitors and also can strongly inhibit ADAM9 protease activity. MMPs were long ago considered as promising targets for treating multifactorial diseases such as cancer. However, they are not as desirable in clinical trials because they are associated with musculoskeletal side effects, and that phenomenon may be induced by inadvertently targeting protective MMPs [122]. Reasons for failure include lack of inhibitor specificity and insufficient knowledge about the complexity of the MMP function. So far, no ADAM9-specific inhibitor against protease activity has been reported yet that does not influence MMPs in general or other ADAM family members.

### 7.3. Natural Flavonoids

Fisetin, a natural flavonoid, is known for antitumor effects in several cancers. It reduces ADAM9 expression by inducing ERK signaling, and its inhibitory effect on cell migration is reversed by an ERK inhibitor (UO126) in renal cell carcinoma [123]. Galangin, a natural flavonoid from plants, acts as an anti-tumor compound against several cancer types. It decreased the mRNA and protein expression of ADAM9, and its inhibitory effect on cell migration can be reversed by treatment with recombinant human ADAM9 protein in vitro. Similar to fisetin, the mechanism of galangin-mediated down-regulated ADAM9 expression is through increasing the ERK pathway in human glioma cells [124].

Licochalcone A, a potent flavonoid isolated from licorice, possesses anti-angiogenesis and anti-tumor effects. It decreased the protein expression of ADAM9 in a dosage-dependent manner in vitro and further suppressed cell migration and invasion. The combination of Licochalcone A and MEKi (PD98059) has synergistic inhibitory effects on ADAM9 protein expression, migration, and invasion in human glioma cells [125]. Altogether, natural flavonoids have potential antitumor effects, and one potential molecular mechanism is through reducing ADAM9 protein expression.

### 7.4. Sorafenib and Regorafenib

Sorafenib, a multiple receptor tyrosine kinase inhibitor, is a clinical drug for HCC treatment. It has been reported to suppress ADAM9 RNA and protein expression and result in the up-regulation of mMICA for enhancing natural killer cell activity against HCC [80]. Regorafenib, another multi-kinase inhibitor, is effective in patients with sorafenib-resistant HCC and significantly reduced the RNA and protein levels of ADAM9 and ADAM10 for the accumulation of mMICA on the HCC cell membrane. This effect partially explained the clinical superiority of regorafenib over sorafenib [126]. Leukotriene receptor antagonists, pranlukast, and montelukast were reported to inhibit the enzymatic activity of ADAM9 in vitro and enhanced mMICA levels in HCC cells [127].

## 8. Concluding Remarks

In physiological conditions, ADAM9 expresses broadly in the body, but it is also involved in various pathophysiological conditions, including inflammation and degenerative diseases. In recent years, the crucial roles of ADAM9 in tumor proliferation, angiogenesis, metastasis, and immune evasion have been broadly studied. Therefore, targeting ADAM9 might provide a new route of treating inflammatory diseases as well as cancer. Evidence to date supports the use of ADAM9 as a potential biomarker in inflammatory diseases and as a prognostic marker in cancers. This would provide more information for patients regarding outcome predictions. Moreover, currently used therapeutic agents (such as sorafenib and regorafenib) work by reducing ADAM9 levels to enhance cancer treatment. These results suggest new ideas regarding therapeutic strategies that directly target the ADAM9 molecule. Previous studies found that there was no major developmental defect except later retinal disorder in ADAM9 knockout mice. This led to the conclusion that it might be a relatively safe therapeutic target in adult patients. However, in the treatment of ADAM9-related disease, one should consider the specificity of medication targeting ADAM9, because modulating the protective activities of MMPs, in general, can cause side effects. Certainly, more research needs to be done to develop optimal drugs that specifically target the ADAM9 protease activity rather than influencing ADAM9 expression. This would provide more effective therapies for treating ADAM9-mediated diseases.

## Figures and Tables

**Table 1:** Studies of ADAM9 in various cancers.

Type	Role of ADAM9 in Cancer		Reference
Lung Cancer	Clinical Significance	Overexpressed in cancer	[25,26,70,71]
		Negative correlation with OS	[25,70,71,72,73,74]
	Mechanism	ADAM9-tPA-CDCP1-Metastasis	[25]
		ADAM9-ANGPT2-Metastasis	[26]
		ADAM9-IL8/VEGFA-Angiogenesis	[26,72]
Prostate Cancer	Clinical Relevance	Overexpressed in cancer	[75,76]
		Negative correlation with RFS	[75]
	Mechanism	Naa10p-ADAM9-Tumorigenesis/Metastasis	[77]
		ADAM9-Integrin Degradation	[78]
Liver Cancer	Clinical Relevance	Negative correlation with immunotherapy response	[79,80]
	Mechanism	ADAM9-MICA cleavage-immune evasion	[80]
		IL-6-ADAM9-JNK-Metastasis	[81]
Breast Cancer	Clinical Relevance	Overexpressed in cancer	[82]
		Positive correlation with progression	[83]
	Mechanism	NSD2-ADAM9-Tumorigenesis	[84]
Pancreatic Cancer	Clinical Relevance	Overexpressed in cancer	[24,85]
		Positive correlation with progression	[86]
		Negative correlation with OS	[24,85]
	Mechanism	KRAS-ADAM9-Tumorigenesis	[87]
		ADAM9-MEK-ERK-Tumorigenesis	[24]
		Circ-ADAM9-ERK-Tumorigenesis	[88]
Brain Cancer	Clinical Relevance	Overexpressed in cancer	[89,90]
		Negative correlation with OS/PFS	[89,90]
	Mechanism	TNC-ADAM9-Metastasis	[90]

OS, overall survival; PFS, progression-free survival; RFS, relapse-free survival.

## Abbreviations

ADAM9	A disintegrin and a metalloprotease 9
ArgBP2	Sorbin and SH3 domain-containing protein 2
AIPC	Androgen-independent prostate cancer
APP	Amyloid-precursor-protein
CDCP1	CUB domain containing protein 1
COPD	Chronic obstructive pulmonary disease
CRD	Cone-rod dystrophy
ECM	Extracellular matrix
EGF	Epidermal growth factor
EMCV	Encephalomyocarditis virus
EMT	Epithelial-mesenchymal transition
EphB4	Ephrin type-B receptor 4
GBM	Glioblastoma
HB-EGF	Heparin-binding EGF-like growth factor
HCC	Hepatocellular carcinoma
HVR	Hyper-variable region
MDC9	Metalloprotease/disintegrin/cysteine-rich protein 9
MGCs	Multinucleated giant cells
MICA	MHC Class I polypeptide-related sequence A
MiRNA	microRNA
MMP	Matrix metalloproteinase
Naa10p	N-α-Acetyltransferase 10 protein
PC	Proprotein convertase
PMNs	Polymorphonuclear neutrophils
PrPc	Cellular prion protein
SH3	Src-homology 3
SNX	Sorting nexin
TNBC	Triple-negative breast cancer
TNC	Tenascin-C
TNF-α	Tumor necrosis factor-α
tPA	Tissue-type plasminogen activator
VE-cadherin	Vascular endothelial cadherin

## References

[1] Hsia H.E., Tüshaus J., Brummer T., Zheng Y., Scilabra S.D., Lichtenthaler S.F. (2019). Functions of ‘A disintegrin and metalloproteases (ADAMs)’ in the mammalian nervous system. Cell. Mol. Life Sci..

[2] Seals D.F., Courtneidge S.A. (2003). The ADAMs family of metalloproteases: Multidomain proteins with multiple functions. Genes Dev..

[3] Weber S., Saftig P. (2012). Ectodomain shedding and ADAMs in development. Development.

[4] Giebeler N., Zigrino P. (2016). A Disintegrin and Metalloprotease (ADAM): Historical Overview of Their Functions. Toxins.

[5] Janes P.W., Saha N., Barton W.A., Kolev M.V., Wimmer-Kleikamp S.H., Nievergall E., Blobel C.P., Himanen J.P., Lackmann M., Nikolov D.B. (2005). Adam meets Eph: An ADAM substrate recognition module acts as a molecular switch for ephrin cleavage in trans. Cell.

[6] Matthews A.L., Noy P.J., Reyat J.S., Tomlinson M.G. (2017). Regulation of A disintegrin and metalloproteinase (ADAM) family sheddases ADAM10 and ADAM17: The emerging role of tetraspanins and rhomboids. Platelets.

[7] Edwards D.R., Handsley M.M., Pennington C.J. (2008). The ADAM metalloproteinases. Mol. Asp. Med..

[8] Lambrecht B.N., Vanderkerken M., Hammad H. (2018). The emerging role of ADAM metalloproteinases in immunity. Nat. Rev. Immunol..

[9] English W.R., Siviter R.J., Hansen M., Murphy G. (2017). ADAM9 is present at endothelial cell-cell junctions and regulates monocyte-endothelial transmigration. Biochem. Biophys. Res. Commun..

[10] Weskamp G., Krätzschmar J., Reid M.S., Blobel C.P. (1996). MDC9, a widely expressed cellular disintegrin containing cytoplasmic SH3 ligand domains. J. Cell Biol..

[11] Rinchai D., Kewcharoenwong C., Kessler B., Lertmemongkolchai G., Chaussabel D. (2015). Increased abundance of ADAM9 transcripts in the blood is associated with tissue damage. F1000Res.

[12] Namba K., Nishio M., Mori K., Miyamoto N., Tsurudome M., Ito M., Kawano M., Uchida A., Ito Y. (2001). Involvement of ADAM9 in multinucleated giant cell formation of blood monocytes. Cell. Immunol..

[13] Wang X., Polverino F., Rojas-Quintero J., Zhang D., Sánchez J., Yambayev I., Lindqvist E., Virtala R., Djukanovic R., Davies D.E. (2018). A Disintegrin and A Metalloproteinase-9 (ADAM9): A Novel Proteinase Culprit with Multifarious Contributions to COPD. Am. J. Respir. Crit. Care Med..

[14] Roychaudhuri R., Hergrueter A.H., Polverino F., Laucho-Contreras M.E., Gupta K., Borregaard N., Owen C.A. (2014). ADAM9 is a novel product of polymorphonuclear neutrophils: Regulation of expression and contributions to extracellular matrix protein degradation during acute lung injury. J. Immunol..

[15] Zigrino P., Steiger J., Fox J.W., Löffek S., Schild A., Nischt R., Mauch C. (2007). Role of ADAM-9 disintegrin-cysteine-rich domains in human keratinocyte migration. J. Biol. Chem..

[16] Abety A.N., Fox J.W., Schönefuß A., Zamek J., Landsberg J., Krieg T., Blobel C., Mauch C., Zigrino P. (2012). Stromal fibroblast-specific expression of ADAM-9 modulates proliferation and apoptosis in melanoma cells in vitro and in vivo. J. Investig. Dermatol..

[17] Dreymueller D., Uhlig S., Ludwig A. (2015). ADAM-family metalloproteinases in lung inflammation: Potential therapeutic targets. Am. J. Physiol. Lung Cell. Mol. Physiol..

[18] Fu Q., Cheng J., Zhang J., Zhang Y., Chen X., Luo S., Xie J. (2017). miR-20b reduces 5-FU resistance by suppressing the ADAM9/EGFR signaling pathway in colon cancer. Oncol. Rep..

[19] Mahimkar R.M., Visaya O., Pollock A.S., Lovett D.H. (2005). The disintegrin domain of ADAM9: A ligand for multiple beta1 renal integrins. Biochem. J..

[20] Shen G., Sun Q., Yao Y., Li S., Liu G., Yuan C., Li H., Xu Y., Wang H. (2020). Role of ADAM9 and miR-126 in the development of abdominal aortic aneurysm. Atherosclerosis.

[21] Zhang P., Shen M., Fernandez-Patron C., Kassiri Z. (2016). ADAMs family and relatives in cardiovascular physiology and pathology. J. Mol. Cell. Cardiol..

[22] Fadl N.N., Ahmed H.H., Booles H.F., Sayed A.H. (2013). Serrapeptase and nattokinase intervention for relieving Alzheimer’s disease pathophysiology in rat model. Hum. Exp. Toxicol..

[23] Cho C. (2012). Testicular and epididymal ADAMs: Expression and function during fertilization. Nat. Rev. Urol..

[24] Grützmann R., Lüttges J., Sipos B., Ammerpohl O., Dobrowolski F., Alldinger I., Kersting S., Ockert D., Koch R., Kalthoff H. (2004). ADAM9 expression in pancreatic cancer is associated with tumour type and is a prognostic factor in ductal adenocarcinoma. Br. J. Cancer.

[25] Lin C.Y., Chen H.J., Huang C.C., Lai L.C., Lu T.P., Tseng G.C., Kuo T.T., Kuok Q.Y., Hsu J.L., Sung S.Y. (2014). ADAM9 promotes lung cancer metastases to brain by a plasminogen activator-based pathway. Cancer Res..

[26] Lin C.Y., Cho C.F., Bai S.T., Liu J.P., Kuo T.T., Wang L.J., Lin Y.S., Lin C.C., Lai L.C., Lu T.P. (2017). ADAM9 promotes lung cancer progression through vascular remodeling by VEGFA, ANGPT2, and PLAT. Sci. Rep..

[27] Schouten L.R., Helmerhorst H.J., Wagenaar G.T., Haltenhof T., Lutter R., Roelofs J.J., van Woensel J.B., van Kaam A.H., Bos A.P., Schultz M.J. (2016). Age-Dependent Changes in the Pulmonary Renin-Angiotensin System Are Associated With Severity of Lung Injury in a Model of Acute Lung Injury in Rats. Crit. Care Med..

[28] Mauch C., Zamek J., Abety A.N., Grimberg G., Fox J.W., Zigrino P. (2010). Accelerated wound repair in ADAM-9 knockout animals. J. Investig. Dermatol..

[29] Mehta V., Fields L., Evans I.M., Yamaji M., Pellet-Many C., Jones T., Mahmoud M., Zachary I. (2018). VEGF (Vascular Endothelial Growth Factor) Induces NRP1 (Neuropilin-1) Cleavage via ADAMs (a Disintegrin and Metalloproteinase) 9 and 10 to Generate Novel Carboxy-Terminal NRP1 Fragments That Regulate Angiogenic Signaling. Arter. Thromb. Vasc. Biol..

[30] Moss M.L., Powell G., Miller M.A., Edwards L., Qi B., Sang Q.X., De Strooper B., Tesseur I., Lichtenthaler S.F., Taverna M. (2011). ADAM9 inhibition increases membrane activity of ADAM10 and controls α-secretase processing of amyloid precursor protein. J. Biol. Chem..

[31] Asai M., Hattori C., Szabó B., Sasagawa N., Maruyama K., Tanuma S., Ishiura S. (2003). Putative function of ADAM9, ADAM10, and ADAM17 as APP alpha-secretase. Biochem. Biophys. Res. Commun..

[32] Horiuchi K., Zhou H.M., Kelly K., Manova K., Blobel C.P. (2005). Evaluation of the contributions of ADAMs 9, 12, 15, 17, and 19 to heart development and ectodomain shedding of neuregulins beta1 and beta2. Dev. Biol..

[33] Sammel M., Peters F., Lokau J., Scharfenberg F., Werny L., Linder S., Garbers C., Rose-John S., Becker-Pauly C. (2019). Differences in Shedding of the Interleukin-11 Receptor by the Proteases ADAM9, ADAM10, ADAM17, Meprin α, Meprin β and MT1-MMP. Int. J. Mol. Sci..

[34] Weskamp G., Cai H., Brodie T.A., Higashyama S., Manova K., Ludwig T., Blobel C.P. (2002). Mice lacking the metalloprotease-disintegrin MDC9 (ADAM9) have no evident major abnormalities during development or adult life. Mol. Cell. Biol..

[35] Goldstein O., Mezey J.G., Boyko A.R., Gao C., Wang W., Bustamante C.D., Anguish L.J., Jordan J.A., Pearce-Kelling S.E., Aguirre G.D. (2010). An ADAM9 mutation in canine cone-rod dystrophy 3 establishes homology with human cone-rod dystrophy 9. Mol. Vis..

[36] Parry D.A., Toomes C., Bida L., Danciger M., Towns K.V., McKibbin M., Jacobson S.G., Logan C.V., Ali M., Bond J. (2009). Loss of the metalloprotease ADAM9 leads to cone-rod dystrophy in humans and retinal degeneration in mice. Am. J. Hum. Genet..

[37] Guaiquil V., Swendeman S., Yoshida T., Chavala S., Campochiaro P.A., Blobel C.P. (2009). ADAM9 is involved in pathological retinal neovascularization. Mol. Cell. Biol..

[38] Moss M.L., Bomar M., Liu Q., Sage H., Dempsey P., Lenhart P.M., Gillispie P.A., Stoeck A., Wildeboer D., Bartsch J.W. (2007). The ADAM10 prodomain is a specific inhibitor of ADAM10 proteolytic activity and inhibits cellular shedding events. J. Biol. Chem..

[39] Gonzales P.E., Solomon A., Miller A.B., Leesnitzer M.A., Sagi I., Milla M.E. (2004). Inhibition of the tumor necrosis factor-α-converting enzyme by its pro domain. J. Biol. Chem..

[40] Wong E., Maretzky T., Peleg Y., Blobel C.P., Sagi I. (2015). The Functional Maturation of A Disintegrin and Metalloproteinase (ADAM) 9, 10, and 17 Requires Processing at a Newly Identified Proprotein Convertase (PC) Cleavage Site. J. Biol. Chem..

[41] Wichert R., Scharfenberg F., Colmorgen C., Koudelka T., Schwarz J., Wetzel S., Potempa B., Potempa J., Bartsch J.W., Sagi I. (2019). Meprin β induces activities of A disintegrin and metalloproteinases 9, 10, and 17 by specific prodomain cleavage. FASEB J..

[42] Smith K.M., Gaultier A., Cousin H., Alfandari D., White J.M., DeSimone D.W. (2002). The cysteine-rich domain regulates ADAM protease function in vivo. J. Cell Biol..

[43] Gaultier A., Cousin H., Darribère T., Alfandari D. (2002). ADAM13 disintegrin and cysteine-rich domains bind to the second heparin-binding domain of fibronectin. J. Biol. Chem..

[44] Kleino I., Järviluoma A., Hepojoki J., Huovila A.P., Saksela K. (2015). Preferred SH3 domain partners of ADAM metalloproteases include shared and ADAM-specific SH3 interactions. PLoS ONE.

[45] Soulet F., Yarar D., Leonard M., Schmid S.L. (2005). SNX9 regulates dynamin assembly and is required for efficient clathrin-mediated endocytosis. Mol. Biol. Cell.

[46] Mygind K.J., Störiko T., Freiberg M.L., Samsøe-Petersen J., Schwarz J., Andersen O.M., Kveiborg M. (2018). Sorting nexin 9 (SNX9) regulates levels of the transmembrane ADAM9 at the cell surface. J. Biol. Chem..

[47] Izumi Y., Hirata M., Hasuwa H., Iwamoto R., Umata T., Miyado K., Tamai Y., Kurisaki T., Sehara-Fujisawa A., Ohno S. (1998). A metalloprotease-disintegrin, MDC9/meltrin-gamma/ADAM9 and PKCdelta are involved in TPA-induced ectodomain shedding of membrane-anchored heparin-binding EGF-like growth factor. EMBO J..

[48] Fry J.L., Toker A. (2010). Secreted and membrane-bound isoforms of protease ADAM9 have opposing effects on breast cancer cell migration. Cancer Res..

[49] Mazzocca A., Coppari R., De Franco R., Cho J.Y., Libermann T.A., Pinzani M., Toker A. (2005). A secreted form of ADAM9 promotes carcinoma invasion through tumor-stromal interactions. Cancer Res..

[50] Seegar T.C., Killingsworth L.B., Saha N., Meyer P.A., Patra D., Zimmerman B., Janes P.W., Rubinstein E., Nikolov D.B., Skiniotis G.J.C. (2017). Structural basis for regulated proteolysis by the α-secretase ADAM10. Cell.

[51] Grötzinger J., Lorenzen I., Düsterhöft S. (2017). Molecular insights into the multilayered regulation of ADAM17: The role of the extracellular region. Biochim. Biophys. Acta (BBA) Mol. Cell Res..

[52] Takeda S., Igarashi T., Mori H., Araki S. (2006). Crystal structures of VAP1 reveal ADAMs’ MDC domain architecture and its unique C-shaped scaffold. EMBO J..

[53] Igarashi T., Araki S., Mori H., Takeda S. (2007). Crystal structures of catrocollastatin/VAP2B reveal a dynamic, modular architecture of ADAM/adamalysin/reprolysin family proteins. FEBS Lett..

[54] Maretzky T., Swendeman S., Mogollon E., Weskamp G., Sahin U., Reiss K., Blobel C.P. (2017). Characterization of the catalytic properties of the membrane-anchored metalloproteinase ADAM9 in cell-based assays. Biochem. J..

[55] Mullooly M., McGowan P.M., Crown J., Duffy M.J. (2016). The ADAMs family of proteases as targets for the treatment of cancer. Cancer Biol. Ther..

[56] Mohan S., Thompson G.R., Amaar Y.G., Hathaway G., Tschesche H., Baylink D. (2002). ADAM-9 is an insulin-like growth factor binding protein-5 protease produced and secreted by human osteoblasts. Biochemistry.

[57] Dyczynska E., Sun D., Yi H., Sehara-Fujisawa A., Blobel C.P., Zolkiewska A. (2007). Proteolytic processing of delta-like 1 by ADAM proteases. J. Biol. Chem..

[58] English W.R., Corvol P., Murphy G. (2012). LPS activates ADAM9 dependent shedding of ACE from endothelial cells. Biochem. Biophys. Res. Commun..

[59] Caescu C.I., Jeschke G.R., Turk B. (2009). Active-site determinants of substrate recognition by the metalloproteinases TACE and ADAM10. Biochem. J..

[60] Ray B., Maloney B., Sambamurti K., Karnati H.K., Nelson P.T., Greig N.H., Lahiri D.K. (2020). Rivastigmine modifies the α-secretase pathway and potentially early Alzheimer’s disease. Transl. Psychiatry.

[61] Sennvik K., Fastbom J., Blomberg M., Wahlund L.O., Winblad B., Benedikz E. (2000). Levels of alpha- and beta-secretase cleaved amyloid precursor protein in the cerebrospinal fluid of Alzheimer’s disease patients. Neurosci. Lett..

[62] Cong L., Jia J. (2011). Promoter polymorphisms which regulate ADAM9 transcription are protective against sporadic Alzheimer’s disease. Neurobiol. Aging.

[63] Guaiquil V.H., Hewing N.J., Chiang M.F., Rosenblatt M.I., Chan R.V., Blobel C.P. (2013). A murine model for retinopathy of prematurity identifies endothelial cell proliferation as a potential mechanism for plus disease. Investig. Ophthalmol. Vis. Sci..

[64] Opdenakker G., Abu El-Asrar A. (2019). Metalloproteinases mediate diabetes-induced retinal neuropathy and vasculopathy. Cell. Mol. Life Sci..

[65] Folkesson M., Li C., Frebelius S., Swedenborg J., Wågsäter D., Williams K.J., Eriksson P., Roy J., Liu M.L. (2015). Proteolytically active ADAM10 and ADAM17 carried on membrane microvesicles in human abdominal aortic aneurysms. Thromb. Haemost..

[66] Geng L., Wang W., Chen Y., Cao J., Lu L., Chen Q., He R., Shen W. (2010). Elevation of ADAM10, ADAM17, MMP-2 and MMP-9 expression with media degeneration features CaCl2-induced thoracic aortic aneurysm in a rat model. Exp. Mol. Pathol..

[67] Yu J., Liu R., Huang J., Wang L., Wang W. (2017). Inhibition of Phosphatidylinositol 3-kinease suppresses formation and progression of experimental abdominal aortic aneurysms. Sci. Rep..

[68] Baggen J., Thibaut H.J., Hurdiss D.L., Wahedi M., Marceau C.D., van Vliet A.L.W., Carette J.E., van Kuppeveld F.J.M. (2019). Identification of the Cell-Surface Protease ADAM9 as an Entry Factor for Encephalomyocarditis Virus. mBio.

[69] Bazzone L.E., King M., MacKay C.R., Kyawe P.P., Meraner P., Lindstrom D., Rojas-Quintero J., Owen C.A., Wang J.P., Brass A.L. (2019). A Disintegrin and Metalloproteinase 9 Domain (ADAM9) Is a Major Susceptibility Factor in the Early Stages of Encephalomyocarditis Virus Infection. mBio.

[70] Liu R., Wang F., Guo Y., Yang J., Chen S., Gao X., Wang X. (2018). MicroRNA-425 promotes the development of lung adenocarcinoma via targeting A disintegrin and metalloproteinases 9 (ADAM9). Onco Targets Ther..

[71] Chang J.H., Lai S.L., Chen W.S., Hung W.Y., Chow J.M., Hsiao M., Lee W.J., Chien M.H. (2017). Quercetin suppresses the metastatic ability of lung cancer through inhibiting Snail-dependent Akt activation and Snail-independent ADAM9 expression pathways. Biochim. Biophys. Acta Mol. Cell Res..

[72] Kossmann C.M., Annereau M., Thomas-Schoemann A., Nicco-Overney C., Chéreau C., Batteux F., Alexandre J., Lemare F. (2017). ADAM9 expression promotes an aggressive lung adenocarcinoma phenotype. Tumour Biol..

[73] Zhang J., Qi J., Chen N., Fu W., Zhou B., He A. (2013). High expression of a disintegrin and metalloproteinase-9 predicts a shortened survival time in completely resected stage I non-small cell lung cancer. Oncol. Lett..

[74] Zhang J., Chen N., Qi J., Zhou B., Qiu X. (2014). HDGF and ADAM9 are novel molecular staging biomarkers, prognostic biomarkers and predictive biomarkers for adjuvant chemotherapy in surgically resected stage I non-small cell lung cancer. J. Cancer Res. Clin. Oncol..

[75] Fritzsche F.R., Jung M., Tölle A., Wild P., Hartmann A., Wassermann K., Rabien A., Lein M., Dietel M., Pilarsky C. (2008). ADAM9 expression is a significant and independent prognostic marker of PSA relapse in prostate cancer. Eur. Urol..

[76] Hua Y., Liang C., Miao C., Wang S., Su S., Shao P., Liu B., Bao M., Zhu J., Xu A. (2018). MicroRNA-126 inhibits proliferation and metastasis in prostate cancer via regulation of ADAM9. Oncol. Lett..

[77] Lin Y.-W., Wen Y.-C., Chu C.-Y., Tung M.-C., Yang Y.-C., Hua K.-T., Pan K.-F., Hsiao M., Lee W.-J., Chien M.-H. (2020). Stabilization of ADAM9 by N-α-acetyltransferase 10 protein contributes to promoting progression of androgen-independent prostate cancer. Cell Death Dis..

[78] Mygind K.J., Schwarz J., Sahgal P., Ivaska J., Kveiborg M. (2018). Loss of ADAM9 expression impairs β1 integrin endocytosis, focal adhesion formation and cancer cell migration. J. Cell Sci..

[79] Oh S., Park Y., Lee H.J., Lee J., Lee S.H., Baek Y.S., Chun S.K., Lee S.M., Kim M., Chon Y.E. (2020). A Disintegrin and Metalloproteinase 9 (ADAM9) in Advanced Hepatocellular Carcinoma and Their Role as a Biomarker During Hepatocellular Carcinoma Immunotherapy. Cancers.

[80] Kohga K., Takehara T., Tatsumi T., Ishida H., Miyagi T., Hosui A., Hayashi N. (2010). Sorafenib inhibits the shedding of major histocompatibility complex class I-related chain A on hepatocellular carcinoma cells by down-regulating a disintegrin and metalloproteinase 9. Hepatology.

[81] Dong Y., Wu Z., He M., Chen Y., Chen Y., Shen X., Zhao X., Zhang L., Yuan B., Zeng Z. (2018). ADAM9 mediates the interleukin-6-induced Epithelial-Mesenchymal transition and metastasis through ROS production in hepatoma cells. Cancer Lett..

[82] O’Shea C., McKie N., Buggy Y., Duggan C., Hill A.D., McDermott E., O’Higgins N., Duffy M.J. (2003). Expression of ADAM-9 mRNA and protein in human breast cancer. Int. J. Cancer.

[83] Oria V.O., Lopatta P., Schilling O. (2018). The pleiotropic roles of ADAM9 in the biology of solid tumors. Cell. Mol. Life Sci..

[84] Wang J.J., Zou J.X., Wang H., Duan Z.J., Wang H.B., Chen P., Liu P.Q., Xu J.Z., Chen H.W. (2019). Histone methyltransferase NSD2 mediates the survival and invasion of triple-negative breast cancer cells via stimulating ADAM9-EGFR-AKT signaling. Acta Pharmacol. Sin..

[85] van Kampen J.G.M., van Hooij O., Jansen C.F., Smit F.P., van Noort P.I., Schultz I., Schaapveld R.Q.J., Schalken J.A., Verhaegh G.W. (2017). miRNA-520f Reverses Epithelial-to-Mesenchymal Transition by Targeting ADAM9 and TGFBR2. Cancer Res..

[86] Oria V.O., Lopatta P., Schmitz T., Preca B.T., Nyström A., Conrad C., Bartsch J.W., Kulemann B., Hoeppner J., Maurer J. (2019). ADAM9 contributes to vascular invasion in pancreatic ductal adenocarcinoma. Mol. Oncol..

[87] Yuan P., He X.H., Rong Y.F., Cao J., Li Y., Hu Y.P., Liu Y., Li D., Lou W., Liu M.F. (2017). KRAS/NF-κB/YY1/miR-489 Signaling Axis Controls Pancreatic Cancer Metastasis. Cancer Res..

[88] Xing C., Ye H., Wang W., Sun M., Zhang J., Zhao Z., Jiang G. (2019). Circular RNA ADAM9 facilitates the malignant behaviours of pancreatic cancer by sponging miR-217 and upregulating PRSS3 expression. Artif. Cells Nanomed. Biotechnol..

[89] Fan X., Wang Y., Zhang C., Liu L., Yang S., Wang Y., Liu X., Qian Z., Fang S., Qiao H. (2016). ADAM9 Expression Is Associate with Glioma Tumor Grade and Histological Type, and Acts as a Prognostic Factor in Lower-Grade Gliomas. Int. J. Mol. Sci..

[90] Sarkar S., Zemp F.J., Senger D., Robbins S.M., Yong V.W. (2015). ADAM-9 is a novel mediator of tenascin-C-stimulated invasiveness of brain tumor-initiating cells. Neuro Oncol..

[91] Shintani Y., Higashiyama S., Ohta M., Hirabayashi H., Yamamoto S., Yoshimasu T., Matsuda H., Matsuura N. (2004). Overexpression of ADAM9 in Non-Small Cell Lung Cancer Correlates with Brain Metastasis. Cancer Res..

[92] Chiu K.L., Kuo T.T., Kuok Q.Y., Lin Y.S., Hua C.H., Lin C.Y., Su P.Y., Lai L.C., Sher Y.P. (2015). ADAM9 enhances CDCP1 protein expression by suppressing miR-218 for lung tumor metastasis. Sci. Rep..

[93] Chiu K.L., Lin Y.S., Kuo T.T., Lo C.C., Huang Y.K., Chang H.F., Chuang E.Y., Lin C.C., Cheng W.C., Liu Y.N. (2017). ADAM9 enhances CDCP1 by inhibiting miR-1 through EGFR signaling activation in lung cancer metastasis. Oncotarget.

[94] Wang F.F., Wang S., Xue W.H., Cheng J.L. (2016). microRNA-590 suppresses the tumorigenesis and invasiveness of non-small cell lung cancer cells by targeting ADAM9. Mol. Cell. Biochem..

[95] Wan J., Hao L., Zheng X., Li Z. (2019). Circular RNA circ_0020123 promotes non-small cell lung cancer progression by acting as a ceRNA for miR-488-3p to regulate ADAM9 expression. Biochem. Biophys. Res. Commun..

[96] Zhang J., Pan Y.F., Ding Z.W., Yang G.Z., Tan Y.X., Yang C., Jiang T.Y., Liu L.J., Zhang B., Han T. (2015). RMP promotes venous metastases of hepatocellular carcinoma through promoting IL-6 transcription. Oncogene.

[97] Xiang L.Y., Ou H.H., Liu X.C., Chen Z.J., Li X.H., Huang Y., Yang D.H. (2017). Loss of tumor suppressor miR-126 contributes to the development of hepatitis B virus-related hepatocellular carcinoma metastasis through the upregulation of ADAM9. Tumour Biol..

[98] Wan D., Shen S., Fu S., Preston B., Brandon C., He S., Shen C., Wu J., Wang S., Xie W. (2016). miR-203 suppresses the proliferation and metastasis of hepatocellular carcinoma by targeting oncogene ADAM9 and oncogenic long non-coding RNA HULC. Anticancer Agents Med. Chem..

[99] Hu D., Shen D., Zhang M., Jiang N., Sun F., Yuan S., Wan K. (2017). MiR-488 suppresses cell proliferation and invasion by targeting ADAM9 and lncRNA HULC in hepatocellular carcinoma. Am. J. Cancer Res..

[100] Micocci K.C., Moritz M.N., Lino R.L., Fernandes L.R., Lima A.G., Figueiredo C.C., Morandi V., Selistre-de-Araujo H.S. (2016). ADAM9 silencing inhibits breast tumor cells transmigration through blood and lymphatic endothelial cells. Biochimie.

[101] Zhu N., Zhang D., Xie H., Zhou Z., Chen H., Hu T., Bai Y., Shen Y., Yuan W., Jing Q. (2011). Endothelial-specific intron-derived miR-126 is down-regulated in human breast cancer and targets both VEGFA and PIK3R2. Mol. Cell. Biochem..

[102] Wang C.Z., Yuan P., Li Y. (2015). MiR-126 regulated breast cancer cell invasion by targeting ADAM9. Int. J. Clin. Exp. Pathol..

[103] Qin C., Zhao Y., Gong C., Yang Z. (2017). MicroRNA-154/ADAM9 axis inhibits the proliferation, migration and invasion of breast cancer cells. Oncol. Lett..

[104] Zhang C., Zhang Y., Ding W., Lin Y., Huang Z., Luo Q. (2015). MiR-33a suppresses breast cancer cell proliferation and metastasis by targeting ADAM9 and ROS1. Protein Cell.

[105] Kristensen L.S., Andersen M.S., Stagsted L.V.W., Ebbesen K.K., Hansen T.B., Kjems J. (2019). The biogenesis, biology and characterization of circular RNAs. Nat. Rev. Genet..

[106] Wu D.M., Wen X., Han X.R., Wang S., Wang Y.J., Shen M., Fan S.H., Zhang Z.F., Shan Q., Li M.Q. (2019). Bone Marrow Mesenchymal Stem Cell-Derived Exosomal MicroRNA-126-3p Inhibits Pancreatic Cancer Development by Targeting ADAM9. Mol. Ther. Nucleic Acids.

[107] Hamada S., Satoh K., Fujibuchi W., Hirota M., Kanno A., Unno J., Masamune A., Kikuta K., Kume K., Shimosegawa T. (2012). MiR-126 acts as a tumor suppressor in pancreatic cancer cells via the regulation of ADAM9. Mol. Cancer Res..

[108] Ji T., Zhang X., Li W. (2017). MicroRNA-543 inhibits proliferation, invasion and induces apoptosis of glioblastoma cells by directly targeting ADAM9. Mol. Med. Rep..

[109] Liu X., Wang S., Yuan A., Yuan X., Liu B. (2016). MicroRNA-140 represses glioma growth and metastasis by directly targeting ADAM9. Oncol. Rep..

[110] Josson S., Anderson C.S., Sung S.Y., Johnstone P.A., Kubo H., Hsieh C.L., Arnold R., Gururajan M., Yates C., Chung L.W. (2011). Inhibition of ADAM9 expression induces epithelial phenotypic alterations and sensitizes human prostate cancer cells to radiation and chemotherapy. Prostate.

[111] Ueno M., Shiomi T., Mochizuki S., Chijiiwa M., Shimoda M., Kanai Y., Kataoka F., Hirasawa A., Susumu N., Aoki D. (2018). ADAM9 is over-expressed in human ovarian clear cell carcinomas and suppresses cisplatin-induced cell death. Cancer Sci..

[112] Amendola R.S., Martin A.C., Selistre-de-Araújo H.S., Paula-Neto H.A., Saldanha-Gama R., Barja-Fidalgo C. (2015). ADAM9 disintegrin domain activates human neutrophils through an autocrine circuit involving integrins and CXCR2. J. Leukoc. Biol..

[113] Yang L., Liu Q., Zhang X., Liu X., Zhou B., Chen J., Huang D., Li J., Li H., Chen F.J.N. (2020). DNA of neutrophil extracellular traps promotes cancer metastasis via CCDC25. Nature.

[114] Puissegur M.-P., Lay G., Gilleron M., Botella L., Nigou J., Marrakchi H., Mari B., Duteyrat J.-L., Guerardel Y., Kremer L. (2007). Mycobacterial lipomannan induces granuloma macrophage fusion via a TLR2-dependent, ADAM9-and β1 integrin-mediated pathway. J. Immunol..

[115] Brooks P.J., Glogauer M., McCulloch C.A. (2019). An overview of the derivation and function of multinucleated giant cells and their role in pathologic processes. Am. J. Pathol..

[116] Nieswandt B., Hafner M., Echtenacher B., Männel D.N. (1999). Lysis of tumor cells by natural killer cells in mice is impeded by platelets. Cancer Res..

[117] Palumbo J.S., Talmage K.E., Massari J.V., La Jeunesse C.M., Flick M.J., Kombrinck K.W., Jirousková M., Degen J.L. (2005). Platelets and fibrin(ogen) increase metastatic potential by impeding natural killer cell-mediated elimination of tumor cells. Blood.

[118] Labelle M., Begum S., Hynes R.O. (2011). Direct signaling between platelets and cancer cells induces an epithelial-mesenchymal-like transition and promotes metastasis. Cancer Cell.

[119] Mammadova-Bach E., Zigrino P., Brucker C., Bourdon C., Freund M., De Arcangelis A., Abrams S.I., Orend G., Gachet C., Mangin P.H. (2016). Platelet integrin α6β1 controls lung metastasis through direct binding to cancer cell-derived ADAM9. JCI Insight.

[120] He B., Zhao Z., Cai Q., Zhang Y., Zhang P., Shi S., Xie H., Peng X., Yin W., Tao Y. (2020). miRNA-based biomarkers, therapies, and resistance in Cancer. Int. J. Biol. Sci..

[121] Kim J.M., Jeung H.C., Rha S.Y., Yu E.J., Kim T.S., Shin Y.K., Zhang X., Park K.H., Park S.W., Chung H.C. (2014). The effect of disintegrin-metalloproteinase ADAM9 in gastric cancer progression. Mol. Cancer Ther..

[122] Overall C.M., Kleifeld O. (2006). Tumour microenvironment-opinion: Validating matrix metalloproteinases as drug targets and anti-targets for cancer therapy. Nat. Rev. Cancer.

[123] Hsieh M.H., Tsai J.P., Yang S.F., Chiou H.L., Lin C.L., Hsieh Y.H., Chang H.R. (2019). Fisetin Suppresses the Proliferation and Metastasis of Renal Cell Carcinoma through Upregulation of MEK/ERK-Targeting CTSS and ADAM9. Cells.

[124] Lei D., Zhang F., Yao D., Xiong N., Jiang X., Zhao H. (2018). Galangin increases ERK1/2 phosphorylation to decrease ADAM9 expression and prevents invasion in A172 glioma cells. Mol. Med. Rep..

[125] Huang C.F., Yang S.F., Chiou H.L., Hsu W.H., Hsu J.C., Liu C.J., Hsieh Y.H. (2018). Licochalcone A inhibits the invasive potential of human glioma cells by targeting the MEK/ERK and ADAM9 signaling pathways. Food Funct..

[126] Arai J., Goto K., Stephanou A., Tanoue Y., Ito S., Muroyama R., Matsubara Y., Nakagawa R., Morimoto S., Kaise Y. (2018). Predominance of regorafenib over sorafenib: Restoration of membrane-bound MICA in hepatocellular carcinoma cells. J. Gastroenterol. Hepatol..

[127] Arai J., Goto K., Otoyama Y., Nakajima Y., Sugiura I., Kajiwara A., Tojo M., Ichikawa Y., Uozumi S., Shimozuma Y. (2020). Leukotriene receptor antagonists enhance HCC treatment efficacy by inhibiting ADAMs and suppressing MICA shedding. Cancer Immunol. Immunother..

